# Cognitive impairment as a mediator of treatment discontinuation after intracerebral hemorrhage in acute leukemia

**DOI:** 10.3389/fmed.2026.1928027

**Published:** 2026-09-07

**Authors:** Wentao Lai, Guanlin Huang, Min Zou, Jingdong Zhang, Jing Hu

**Affiliations:** 1 Department of Neurosurgery, Ganzhou Hospital-Nanfang Hospital, Southern Medical University, Ganzhou, China; 2 Department of Neurosurgery, Ganzhou People's Hospital, Ganzhou, China; 3 Department of Hematology, Ganzhou Hospital-Nanfang Hospital, Southern Medical University, Ganzhou, China; 4 Department of Hematology, Ganzhou People's Hospital, Ganzhou, China; 5 Jiangxi Health Commission Key Laboratory of Leukemia, the Affiliated Ganzhou Hospital, Jiangxi Medical College, Nanchang University, Ganzhou, China

**Keywords:** acute leukemia, causal mediation analysis, cognitive impairment, intracerebral hemorrhage, modified rankin scale, Montreal cognitive assessment, treatment discontinuation

## Abstract

**Background:**

Neurological deficits after spontaneous intracerebral hemorrhage (ICH) are linked to treatment discontinuation in acute leukemia patients, but underlying mechanisms are unknown. We tested whether cognitive impairment mediates this relationship.

**Methods:**

In this single-center retrospective cohort study (2016–2024), we included 102 acute leukemia patients who survived ≥6 months post-ICH and remained on hematologic treatment at the 6 month landmark. Neurological deficit at discharge [modified Rankin Scale (mRS), 0–2 vs. 3–5] was the exposure; MoCA score at 6 months was the mediator; time to permanent treatment discontinuation (non progression) was the outcome. We applied counterfactual mediation analysis with 1,000 bootstraps, reporting natural direct/indirect effects (NDE/NIE) as hazard ratios (HRs). We tested the proportional hazards assumption, quantified inter-rater agreement for outcome adjudication, and performed sensitivity analyses (continuous exposure, binary mediator, competing risks, and E-value).

**Results:**

Mean MoCA was 22.0 (SD 5.2); 48.0% had impairment (MoCA < 26). Over 12 month median follow up, 44.1% discontinued. Total effect of mRS 3–5 (vs. 0–2) was HR 3.42 (95% CI 2.05–5.72). Decomposition yielded NDE 2.08 (1.13–3.84) and NIE 1.64 (1.17–2.32), with 42.1% (15.4%–68.4%) mediated via cognition. Sensitivity analyses produced consistent estimates (NIE range 1.25–1.55; mediated proportion 35.8%–38.9%); E-value for NIE was 2.66 (lower CI 1.62).

**Conclusions:**

In this cohort of 6-month ICH survivors with acute leukemia, cognitive impairment was estimated to mediate 42.1% of the neurological deficit effect on hematologic treatment discontinuation. These findings are hypothesis-generating and suggest that post-ICH cognitive dysfunction may be a modifiable mechanism. Cognitive screening and early rehabilitation may warrant integration into post-ICH care; prospective trials are needed to test whether cognitive interventions improve treatment persistence.

## 1 Introduction

Acute leukemia patients are at heightened risk of spontaneous intracerebral hemorrhage (ICH) due to severe thrombocytopenia, coagulopathy, endothelial damage from chemotherapy and leukemia cell infiltration (1, 2). Although advances in hematologic and neurocritical care have enabled more patients to survive the acute ICH episode, survivors frequently sustain persistent neurological deficits and cognitive impairment (3). Neurological disability at discharge, commonly quantified by the modified Rankin Scale (mRS), is strongly associated with subsequent inability to tolerate or adhere to curative hematologic treatments (4, 5). However, the mechanisms through which neurological deficits translate into treatment discontinuation remain poorly understood. Among plausible mediators, cognitive impairment is particularly compelling, as post-ICH cognitive dysfunction encompasses deficits in executive function, processing speed, and medical decision-making capacity—domains that directly undermine a patient's ability to comply with complex treatment protocols and shared decision-making (6–8).

To date, studies in the general ICH population have demonstrated that cognitive impairment is prevalent, progressive, and only partially correlated with focal neurological deficits (9). In the acute leukemia context, emerging evidence suggests that survivors of chemotherapy-related neurotoxicity and intracranial events experience a substantial burden of cognitive decline (10, 11), yet the downstream consequences for hematologic treatment trajectories have not been systematically examined. Crucially, no investigation has examined whether cognitive impairment acts as an intermediate pathway linking the severity of post-ICH neurological deficits to the discontinuation of life-saving hematologic therapy (12). Establishing such a pathway would identify cognitive impairment as a potentially modifiable target, distinct from irreversible structural neurological damage, and open a new avenue for cognitive rehabilitation interventions aimed at preserving treatment continuity.

We therefore conducted a retrospective longitudinal cohort study to test whether 6-month cognitive impairment mediates the effect of post-ICH neurological deficits on hematologic treatment discontinuation, independent of disease progression. Using a counterfactual causal mediation framework, we decomposed the total effect into direct and indirect pathways. The novelty is threefold: (i) we empirically test a temporally ordered “neurological injury → cognitive impairment → treatment termination” pathway in acute leukemia survivors with ICH; (ii) we quantify the proportion of the total effect that could theoretically be mitigated by preserving cognitive function; and (iii) our findings suggest that cognitive impairment may operate not merely as a secondary morbidity but as a potentially modifiable mediator of hematologic treatment failure, thereby supporting evaluation of cognitive screening and rehabilitation as components of post-ICH care.

## 2 Methods

### 2.1 Study design and participants

**Figure 1:**
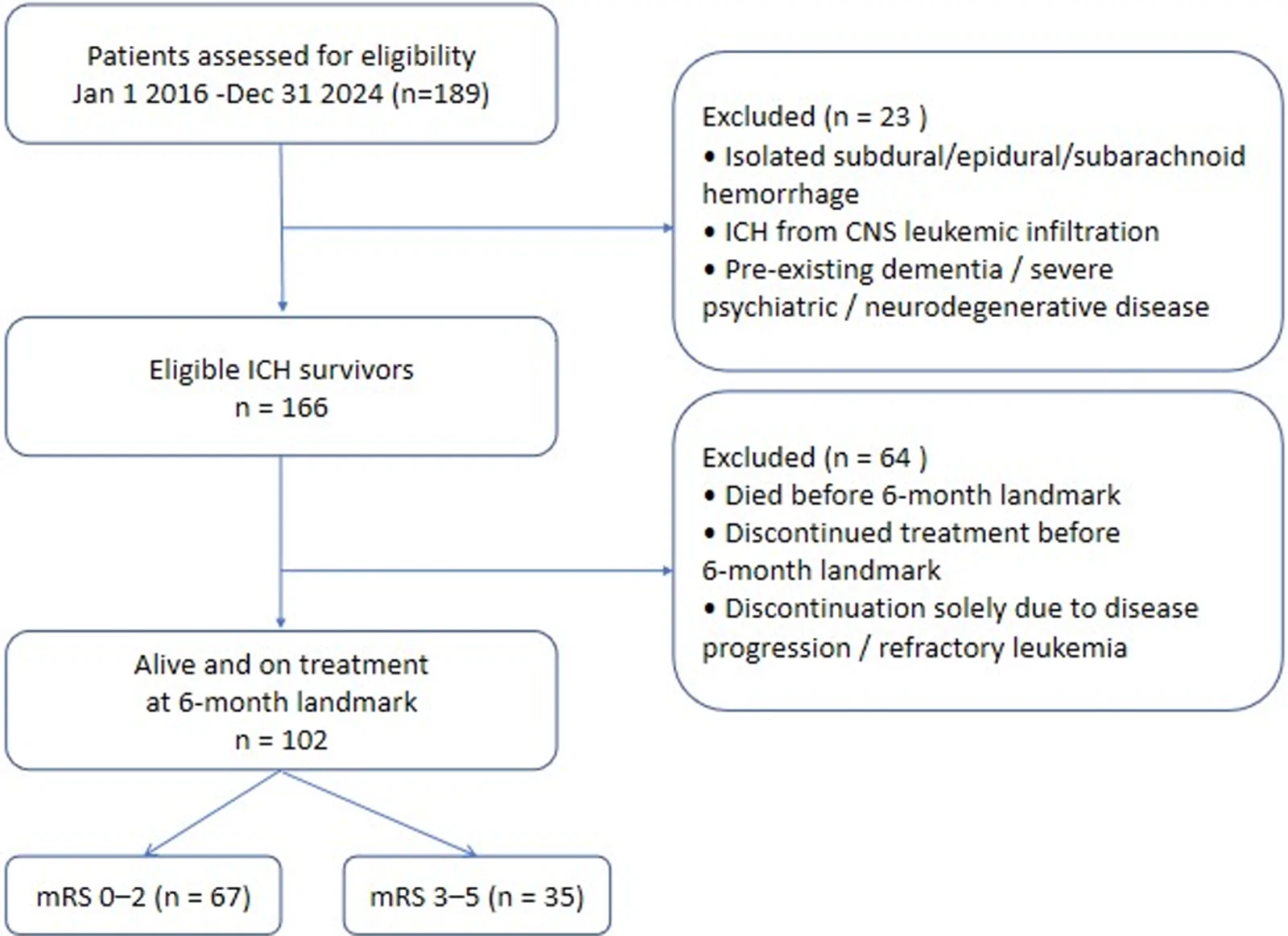
Study flow diagram showing patient screening, exclusions, and the final analytic cohort (*n* = 102).

We conducted a single-center, retrospective cohort study at Ganzhou People's Hospital between January 2016 and December 2024. Consecutive adult patients (≥18 years) with a confirmed diagnosis of acute leukemia [acute myeloid leukemia (AML) or acute lymphoblastic leukemia (ALL)] who developed spontaneous, non-traumatic ICH during active hematologic treatment were screened. The ICH had to be radiologically confirmed by computed tomography or magnetic resonance imaging and localized primarily within the brain parenchyma. We excluded patients with isolated subdural, epidural, or subarachnoid hemorrhage, ICH secondary to central nervous system leukemic infiltration, or pre-existing documented dementia, severe psychiatric disorders, or neurodegenerative disease predating the ICH. To establish the temporally ordered exposure–mediator–outcome sequence required for causal mediation, we further restricted the cohort to patients who survived at least 6 months after the index ICH and remained on hematologic treatment without having discontinued it prior to the 6-month landmark. Patients whose termination of hematologic therapy was exclusively attributable to definitive disease progression or refractory leukemia, as determined by independent adjudication, were excluded. The final analytic cohort comprised 102 patients. (Figure 1) As the cohort is restricted to 6-month survivors still on treatment, our analysis estimates effects within a principal stratum of individuals who would survive and adhere to therapy regardless of neurological deficit severity. The resulting natural direct and indirect effects are interpretable as survivor average causal mediation effects, conditional on this stratum. Extrapolation beyond this group would require additional assumptions. Because neurological deficit severity itself influences early mortality and early treatment cessation, this conditioning induces selection (collider) bias; the estimated effects are therefore conditional on the survivor-and-adherent stratum and cannot be interpreted as applying to the entire population of patients with acute leukemia and ICH. The institutional review board of Ganzhou People's Hospital approved the study with a waiver of informed consent owing to its retrospective nature.

### 2.2 Variables and assessment time points

The three core variables were operationalized along a strict temporal sequence to support causal inference.

Exposure: Neurological deficit severity at hospital discharge (T0). The severity of neurological deficit resulting from the ICH was assessed at the time of hospital discharge using the mRS, a validated 7-point ordinal scale ranging from 0 (no symptoms) to 6 (death). Because the cohort excluded patients who died before the 6-month landmark, the mRS range was 0–5. For primary analyses, the exposure was dichotomized into no/mild deficit (mRS 0–2) versus moderate-to-severe deficit (mRS 3–5). In sensitivity analyses, the mRS was modeled as a continuous variable (0–5) to test linearity assumptions.

Mediator: Cognitive impairment at 6 months post-ICH (T1). Cognitive function was evaluated at 6 months (±1 month) after the index ICH using the Montreal Cognitive Assessment (MoCA), administered by trained neuropsychologists using culturally adapted and education-adjusted versions. The neuropsychologists administering the MoCA were blinded to mRS status and to subsequent treatment outcomes. The MoCA yields a total score of 0–30, with lower scores indicating greater cognitive impairment. The total MoCA score was the primary continuous mediator. In sensitivity analyses, we used a validated cut-off of <26 to define cognitive impairment as a binary mediator.

Outcome: Hematologic treatment discontinuation after T1 (T2). The time-to-event outcome was permanent discontinuation of the originally planned hematologic treatment (intensive chemotherapy, targeted therapy, or hematopoietic stem cell transplantation) for any reason other than documented disease progression. Discontinuation events were identified from medical records by two hematologists blinded to mRS group and MoCA scores, who independently adjudicated each case and classified the primary reason into intolerance/poor performance status, cognitive inability to comply with treatment, or shared decision-making due to multifaceted factors. When multiple reasons contributed, the primary (dominant) reason was assigned using a prespecified hierarchy. Inter-rater agreement between the two adjudicators was high, with Cohen's *κ* of 0.88 (95% CI 0.78–0.98). Discrepancies were resolved by consensus. The time origin was the T1 visit date (6 months post-ICH), and patients were followed until treatment discontinuation, death without prior discontinuation (treated as a competing event), loss to follow-up, or the administrative censoring date of December 31, 2024.

Covariates: The following prespecified covariates measured at or before T0 were included to adjust for potential confounding of the exposure–mediator, exposure–outcome, and mediator–outcome relationships: age (continuous), sex, education years (continuous), leukemia type (AML vs. ALL), leukemia risk stratification (favorable, intermediate, adverse), treatment phase at ICH (induction, consolidation, maintenance), ICH characteristics [hematoma volume as a continuous variable, location categorized as deep/lobar/infratentorial, intraventricular extension, and lowest Glasgow Coma Scale (GCS) score], Charlson Comorbidity Index, prior cerebrovascular disease, admission platelet count, and use of anticoagulant or antiplatelet agents.

### 2.3 Causal mediation analysis

There were no missing data for the exposure, mediator, outcome, and covariates in the primary analysis; consequently, a complete-case analysis was performed on all 102 patients. Descriptive statistics were compared between mRS 0–2 and mRS 3–5 groups using t-tests, Mann–Whitney U tests, *χ*^2^ tests, or Fisher's exact tests as appropriate.

We applied the counterfactual-based causal mediation framework for time-to-event outcomes proposed by Valeri and VanderWeele (13) to decompose the total effect (TE) of neurological deficit severity on treatment discontinuation into a natural direct effect (NDE) and a natural indirect effect (NIE) mediated through MoCA score. The mediator model was a linear regression with MoCA score as the dependent variable and mRS group plus covariates as independent variables. The outcome model was a Cox proportional hazards model for time to treatment discontinuation with mRS group, MoCA score (continuous), and covariates as predictors. To balance confounding control against the risk of model instability given the modest number of events (*n* = 45), the primary models included a parsimonious set of five covariates chosen *a priori* on clinical grounds (age, education, hematoma volume, leukemia type, and treatment phase at ICH); the full list of candidate covariates considered is described in Section 2.2, and Table 1 shows that the mRS groups were otherwise well balanced across the remaining covariates. The proportional hazards assumption underlying the Cox model was evaluated using scaled Schoenfeld residuals. The primary analysis used a standard Cox model with death without prior discontinuation treated as censored, because treatment discontinuation (rather than death) was the event of etiologic interest and death was relatively infrequent (*n* = 8; 7.8%); the competing-risk (Fine-Gray) analysis confirms that treating death as a competing event did not materially alter the indirect effect. From these two models, we simulated 1,000 bootstrap resamples to obtain bias-corrected 95% confidence intervals (CIs) for the NDE, NIE, and TE, expressed as hazard ratios (HRs). We then estimated the proportion mediated (PM) on the hazard ratio scale as PM = ln(NIE)/ln(TE), with its 95% CI derived from the bootstrap distribution. The NIE is interpreted as the hazard ratio for treatment discontinuation comparing mRS 3–5 to mRS 0–2 that operates through mRS-associated changes in MoCA score, while the NDE is the hazard ratio through pathways independent of MoCA.

**Table 1:** Baseline characteristics of acute leukemia survivors of ICH at hospital discharge, according to neurological deficit severity.

Characteristic	Overall (*N* = 102)	mRS 0–2 (*n* = 67)	mRS 3–5 (*n* = 35)	*P* value
Demographics				
Age, years, mean ± SD	50.1 ± 14.0	49.5 ± 14.2	51.2 ± 13.8	0.56
Male sex, *n* (%)	58 (56.9)	38 (56.7)	20 (57.1)	0.97
Education, years, median (IQR)	10 (7–12)	10 (8–12)	9 (6–12)	0.18
Leukemia characteristics				
Diagnosis, *n* (%)				0.96
AML	79 (77.5)	52 (77.6)	27 (77.1)	
ALL	23 (22.5)	15 (22.4)	8 (22.9)	
Risk stratification, *n* (%)				0.95
Favorable	15 (14.7)	10 (14.9)	5 (14.3)	
Intermediate	56 (54.9)	37 (55.2)	19 (54.3)	
Adverse	31 (30.4)	20 (29.9)	11 (31.4)	
Treatment phase at ICH, *n* (%)				0.80
Induction	73 (71.6)	47 (70.1)	26 (74.3)	
Consolidation	22 (21.6)	15 (22.4)	7 (20.0)	
Maintenance	7 (6.9)	5 (7.5)	2 (5.7)	
ICH characteristics				
Hematoma volume, mL, median (IQR)	13 (5–20)	9 (2–13)	15 (12–24)	<0.001
Hematoma location, *n* (%)				0.01
Deep	48 (47.1)	30 (44.8)	18 (51.4)	
Lobar	43 (42.2)	33 (49.3)	10 (28.6)	
Infratentorial	11 (10.8)	4 (6.0)	7 (20.0)	
Intraventricular extension, *n* (%)	31 (30.4)	11 (16.4)	20 (57.1)	<0.001
Lowest GCS score, median (IQR)	14 (11–15)	15 (14–15)	11 (8–13)	<0.001
Comorbidity & medications				
Charlson Comorbidity Index, median (IQR)	3 (2–4)	3 (2–4)	3 (2–4)	0.45
Prior cerebrovascular disease, *n* (%)	7 (6.9)	4 (6.0)	3 (8.6)	0.69
Platelet count at admission, × 10⁹/L, median (IQR)	16 (7–27)	18 (8–30)	12 (6–22)	0.08
Anticoagulant/antiplatelet use, *n* (%)	5 (4.9)	3 (4.5)	2 (5.7)	0.67

All variables were assessed at hospital discharge for the index ICH (T0). The cohort comprised patients who survived ≥6 months and had not discontinued hematologic treatment before the 6-month cognitive assessment.

mRS, modified Rankin Scale; ICH, intracerebral hemorrhage; AML, acute myeloid leukemia; ALL, acute lymphoblastic leukemia; GCS, Glasgow Coma Scale.

#### 2.3.1 Causal mediation assumptions

Four key assumptions are required (14):

A1 (No unmeasured exposure–outcome confounding): We adjusted for major pre-ICH predictors; the balance in Table 1 supports this, though residual confounding is possible.

A2 (No unmeasured mediator–outcome confounding): Covariates in the outcome model and an E-value of 2.66 provide some protection, but unmeasured confounders cannot be excluded.

A3 (No unmeasured exposure–mediator confounding): The same pre-exposure covariates were used, but genetic/exercise data were unavailable.

A4 (No exposure-induced mediator–outcome confounding): This is the most vulnerable assumption. New-onset depression, post-discharge complications, changes in caregiver support, or intercurrent illness arising between discharge and the 6-month MoCA assessment could be consequences of the neurological deficit while simultaneously worsening cognition and treatment persistence, thereby confounding the mediator-outcome path and potentially biasing the estimated indirect effect.

### 2.4 Sensitivity analyses

We performed a series of prespecified sensitivity analyses to evaluate the robustness of the mediation findings. First, we replicated the mediation analysis using mRS as a continuous exposure (0–5) instead of dichotomized. Second, we modeled the mediator as a binary variable (MoCA <26 vs. ≥ 26) using a logistic regression for the mediator and a Cox model for the outcome, with a resampling-based approach. Third, we addressed the competing risk of death without prior treatment discontinuation by employing the Fine-Gray subdistribution hazard model for the outcome in the mediation framework, generating subdistribution HRs. Fourth, to quantify the potential impact of unmeasured confounding, we calculated the E-value, which represents the minimum strength of association that an unmeasured confounder would need to have with both the mediator and the outcome to fully explain the observed NIE point estimate or shift its CI to include the null, conditional on measured covariates. Fifth, to address potential circularity between the mediator (MoCA-based cognitive impairment) and the outcome definition, in which “cognitive inability to comply” was one adjudicated reason for discontinuation, we repeated the mediation analysis after excluding patients whose adjudicated discontinuation was attributed to cognitive inability to comply (results in Section 3.4).

### 2.5 Statistical software

All analyses were conducted using R software version 4.3.1 (R Foundation for Statistical Computing, Vienna, Austria). The causal mediation analyses were performed with the CMAverse and mediation packages, and the competing-risk sensitivity analysis with the riskRegression and cmprsk packages.

## 3 Results

### 3.1 Baseline characteristics

The final analytic cohort comprised 102 patients who survived at least 6 months after spontaneous ICH during treatment for acute leukemia and who had not discontinued hematologic therapy before the 6-month landmark. At hospital discharge, 67 patients (65.7%) had no or mild neurological deficit (mRS 0–2) and 35 (34.3%) had moderate-to-severe deficit (mRS 3–5). Table 1 presents the baseline characteristics of the cohort stratified by neurological deficit severity. The mean age was 50.1 years (SD 14.0), 56.9% were male, and 77.5% had acute myeloid leukemia, with the majority (71.6%) receiving induction chemotherapy at the time of ICH. Compared with the mRS 0–2 group, patients with mRS 3–5 had significantly larger hematoma volumes [median 15 mL (IQR 12–24) vs. 9 mL [IQR 2–13]; *P* < 0.001], a higher prevalence of intraventricular extension (57.1% vs. 16.4%; *P* < 0.001), and lower GCS scores [median 11 (IQR 8–13) vs. 15 (IQR 14–15); *P* < 0.001]. The two groups were comparable regarding age, sex, education, leukemia type and risk stratification, comorbidity burden, and baseline platelet count, indicating that residual confounding by these demographic and hematologic factors was unlikely to be substantial.

### 3.2 Cognitive function and subsequent hematologic treatment discontinuation

**Figure 2:**
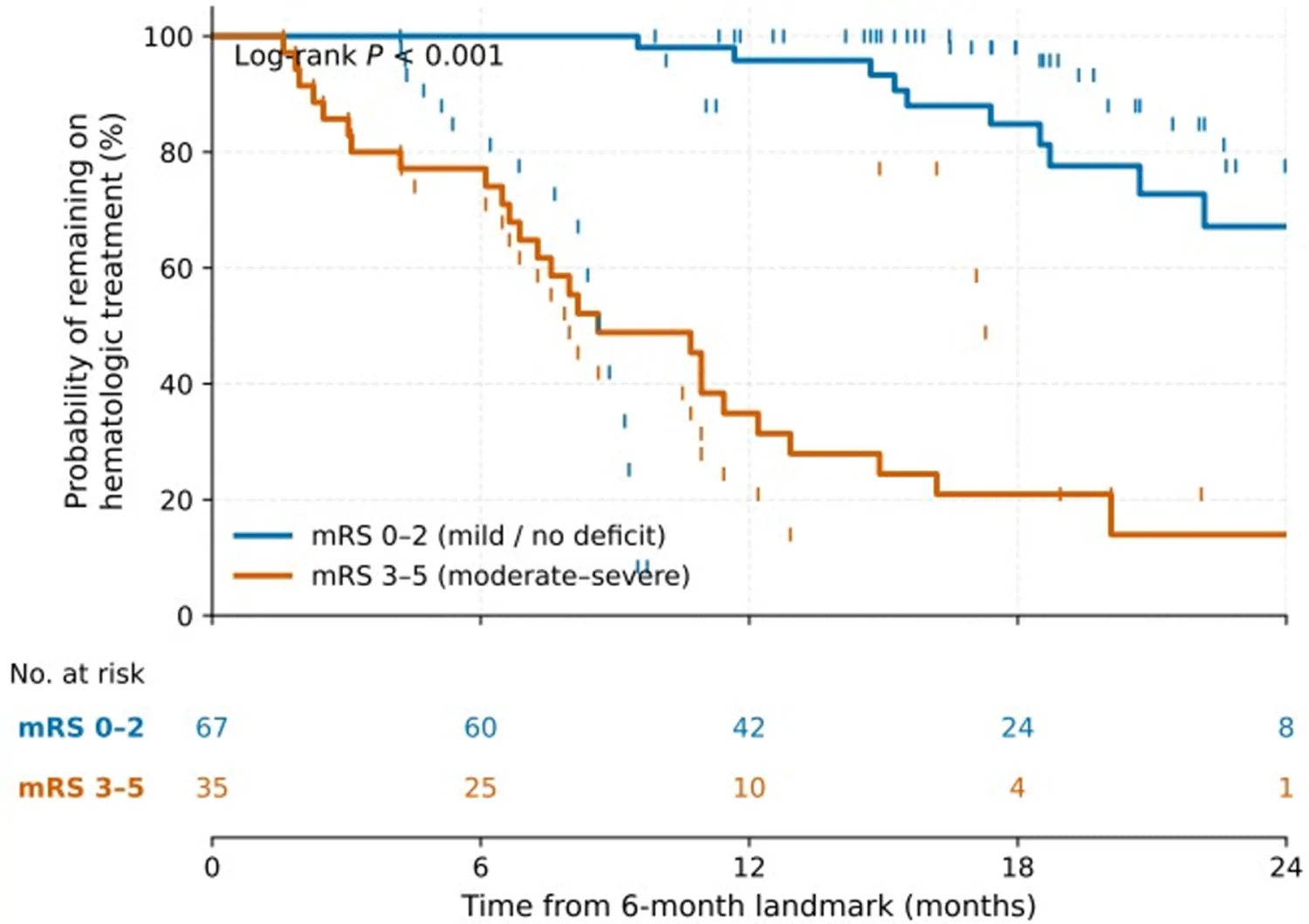
Kaplan–Meier estimates of time to hematologic treatment discontinuation from the 6-month landmark, stratified by neurological deficit severity at discharge (mRS 0-2 vs. mRS 3-5).

At the 6-month post-ICH assessment (T1), the mean MoCA score in the overall cohort was 22.0 (SD 5.2), and 48.0% of patients scored below the standard cut-off of 26 (Table 2). Cognitive impairment was markedly more pronounced in the moderate-to-severe neurological deficit group than in the mild deficit group (mean MoCA: 18.2 ± 4.9 vs. 24.1 ± 4.3; *P* < 0.001; prevalence of MoCA <26: 71.4% vs. 35.8%; *P* < 0.001). Over a median follow-up of 12 months (IQR 6–20) from the 6-month landmark, 45 patients (44.1%) permanently discontinued hematologic treatment for reasons other than disease progression. (Figure 2) The discontinuation rate was substantially higher in the mRS 3–5 group compared with the mRS 0–2 group (77.1% vs. 26.9%; *P* < 0.001), and the median time to discontinuation was shorter [6 months (IQR 3–11) vs. 18 months (IQR 10–26); *P* < 0.001]. Among discontinued patients, cognitive inability to comply with treatment was the adjudicated primary reason for 51.9% in the mRS 3–5 group versus only 11.1% in the mRS 0–2 group. Death without prior discontinuation occurred in 8 patients (7.8%), with no significant difference between groups (6.0% vs. 11.4%; *P* = 0.44). Inter-rater agreement for the adjudication of discontinuation reasons was high, as detailed in the Methods (Cohen's *κ* = 0.88, 95% CI 0.78–0.98).

**Table 2:** Cognitive function at 6-month post-ICH and subsequent hematologic treatment outcomes, overall and by neurological deficit severity at discharge.

Variable	Overall (*N* = 102)	mRS 0–2 (*n* = 67)	mRS 3–5 (*n* = 35)	*P* value
Cognitive assessment at 6 months (T1)				
MoCA total score, mean ± SD	22.0 ± 5.2	24.1 ± 4.3	18.2 ± 4.9	<0.001
MoCA <26, *n* (%)	49 (48.0)	24 (35.8)	25 (71.4)	<0.001
Treatment discontinuation from T1 to end of follow-up				
Discontinued hematologic treatment, *n* (%)	45 (44.1)	18 (26.9)	27 (77.1)	<0.001
Reason for discontinuation, *n* (% of discontinued)				0.001
Intolerance/poor performance status	22 (48.9)	10 (55.6)	12 (44.4)	
Cognitive inability to comply	16 (35.6)	2 (11.1)	14 (51.9)	
Shared decision-making (multiple factors)	7 (15.6)	6 (33.3)	1 (3.7)	
Time to discontinuation, months, median (IQR)	12 (6–20)	18 (10–26)	6 (3–11)	<0.001
Competing events and status at end of follow-up				
Death without prior discontinuation, *n* (%)	8 (7.8)	4 (6.0)	4 (11.4)	0.44
Alive and on hematologic treatment, *n* (%)	49 (48.0)	45 (67.2)	4 (11.4)	<0.001

The cohort comprised patients who survived ≥6 months post-ICH and had not discontinued hematologic treatment before the 6-month cognitive assessment. Follow-up for treatment discontinuation started at T1 (6-month visit). Reasons for discontinuation were independently adjudicated by two reviewers blinded to mRS group; only non-disease-progression causes were counted.

MoCA, Montreal Cognitive Assessment; mRS, modified Rankin Scale; IQR, interquartile range.

### 3.3 Causal mediation analysis

The primary causal mediation analysis for the time-to-event outcome, adjusted for age, education, hematoma volume, leukemia type, and treatment phase, is summarized in Table 3. The total effect of moderate-to-severe neurological deficit (mRS 3–5 vs. 0–2) on hematologic treatment discontinuation corresponded to a hazard ratio (HR) of 3.42 (95% CI 2.05–5.72; *P* < 0.001). Decomposing this total effect, the natural direct effect (NDE), representing pathways not mediated through 6-month MoCA score, was HR 2.08 (95% CI 1.13–3.84; *P* = 0.019). The natural indirect effect (NIE), which quantifies the effect of neurological deficit severity on treatment discontinuation operating through changes in cognitive function, was HR 1.64 (95% CI 1.17–2.32; *P* = 0.004). The proportion mediated, expressed as the fraction of the total effect on the log-hazard scale attributable to cognitive impairment, was 42.1% (95% CI 15.4%–68.4%). These results indicate that nearly half of the excess risk of treatment discontinuation associated with more severe post-ICH neurological deficits was conveyed through worsening cognitive performance. Coefficients from the mediator and outcome models are provided in Supplementary Table S1. The proportional hazards assumption was satisfied (scaled Schoenfeld residuals, all *P* > 0.10; Supplementary Table S1).

**Table 3:** Direct and indirect effects of neurological deficit severity on hematologic treatment discontinuation mediated through cognitive impairment: causal mediation analysis for time-to-event outcome.

Effect	Hazard Ratio (95% CI)	*P* value
Total Effect (TE) — mRS 3–5 vs. 0–2	3.42 (2.05, 5.72)	<0.001
Natural Direct Effect (NDE) — not through MoCA	2.08 (1.13, 3.84)	0.019
Natural Indirect Effect (NIE) — through MoCA score	1.64 (1.17, 2.32)	0.004
Proportion Mediated (PM), % (95% CI)	42.1 (15.4, 68.4)	—

Analyses were based on the counterfactual framework with a Cox proportional hazards model for the outcome and a linear regression model for the mediator, using 1,000 bootstrap resamples. The NIE can be interpreted as the hazard ratio for treatment discontinuation comparing mRS 3–5 to mRS 0–2 that is attributable to the change in MoCA score induced by neurological deficit severity. Models were adjusted for age, education, hematoma volume, leukemia type (AML/ALL), and treatment phase at ICH. The PM was calculated as [ln(NIE)/ln(TE)] on the hazard ratio scale.

mRS, modified Rankin Scale; MoCA, Montreal Cognitive Assessment; CI, confidence interval.

### 3.4 Sensitivity analyses

Multiple sensitivity analyses were conducted to test the robustness of the indirect effect, and all results were consistent with those of the primary analysis (Table 4). When mRS was modeled as a continuous score (per 1-point increase), the NIE remained significant (HR 1.25, 95% CI 1.10–1.42), with a proportion mediated of 35.8% (95% CI 12.1%–58.6%). Dichotomizing the cognitive mediator (MoCA <26 vs. ≥ 26) yielded an NIE of HR 1.48 (95% CI 1.10–1.99) and a proportion mediated of 37.4% (95% CI 10.2%–63.0%). In a competing-risk regression accounting for death without prior treatment discontinuation as a competing event, the indirect effect on the subdistribution hazard of treatment discontinuation was 1.55 (95% CI 1.12–2.15), with 38.9% (95% CI 11.7%–65.5%) of the total effect mediated by cognition. Finally, the E-value for the point estimate of the NIE was 2.66 and the E-value for the lower confidence limit was 1.62, indicating that an unmeasured confounder would need to be associated with both cognitive impairment and treatment discontinuation by a risk ratio of at least 2.66 to fully explain away the observed indirect effect, and at least 1.62 to shift the confidence interval to include the null. In a further sensitivity analysis excluding patients whose discontinuation was adjudicated as “cognitive inability to comply” (*n* = 16), the indirect effect remained statistically significant, with an NIE of 1.33 (95% CI 1.06–1.68) and a proportion mediated of 29.1% (95% CI 6.8%–51.4%). Collectively, these analyses support that the mediating role of cognitive impairment is consistent across alternative specifications and is unlikely to be an artifact of modeling choices or unmeasured confounding of moderate strength.

**Table 4:** Sensitivity analyses for the indirect effect of neurological deficit severity on treatment discontinuation via cognitive impairment.

Analysis	NIE, HR (95% CI)	Proportion Mediated, % (95% CI)
Primary analysis (mRS 0–2 vs. 3–5, MoCA continuous)	1.64 (1.17, 2.32)	42.1 (15.4, 68.4)
Exposure as continuous (mRS per 1-point increase)	1.25 (1.10, 1.42)	35.8 (12.1, 58.6)
Mediator dichotomized (MoCA <26 vs.≥26)	1.48 (1.10, 1.99)	37.4 (10.2, 63.0)
Competing risk model (Fine-Gray, death as competing event)^a^	1.55 (1.12, 2.15)^a^	38.9 (11.7, 65.5)
Unmeasured confounding assessment		
E-value for NIE point estimate (lower 95% CI limit)	2.66 (1.62)	—

All models adjusted for age, education, hematoma volume, leukemia type, and treatment phase, as in the primary analysis. The E-value quantifies the minimum strength of association that an unmeasured confounder would need to have with both the mediator and the outcome to fully explain away the observed NIE point estimate (or shift its CI to include 1), conditional on the measured covariates. These analyses indicate that the cognitive impairment mediation pathway was consistent across alternative specifications.

NIE, natural indirect effect; HR, hazard ratio; CI, confidence interval; mRS, modified Rankin Scale; MoCA, Montreal Cognitive Assessment.

a From Fine-Gray competing-risk regression, the NIE is expressed as a subdistribution hazard ratio—the effect on the instantaneous risk of treatment discontinuation in subjects who have not yet discontinued and who remain alive (i.e., the subdistribution of discontinuation, with death treated as a competing event).

## 4 Discussion

In this cohort of acute leukemia survivors with spontaneous ICH, we tested whether 6-month cognitive impairment mediates the effect of acute neurological deficits on hematologic treatment discontinuation. Within the principal stratum of 6-month survivors still on treatment, cognitive dysfunction mediated 42.1% of the total effect of neurological deficit severity on treatment termination. These findings provide, to our knowledge, the first quantitative evidence that cognition may represent a potentially modifiable conduit through which structural brain injury disrupts oncologic care. The indirect effect remained consistent across multiple sensitivity analyses, including alternative exposure and mediator definitions, competing risk models, and E-value assessments.

Our findings extend the existing literature in two important respects. First, the prevalence of cognitive impairment in this cohort was substantial: 48.0% overall and 71.4% among patients with moderate-to-severe neurological deficits (mRS 3–5). These rates are notably higher than those reported in unselected ICH populations. A systematic review and meta-analysis by Kazim et al. (7) involving 3,270 ICH survivors found a pooled prevalence of post-ICH cognitive impairment of 46% (95% CI, 35.9%–55.9%), with estimates ranging from 35% (long-term follow-up) to 55% (within six months). Similarly, Rost et al. (6) noted that the reported prevalence of post-stroke cognitive impairment varies widely depending on stroke type, definition, and assessment timing, with some studies documenting rates exceeding 70% within three months after ICH. The higher burden observed in our cohort likely reflects the unique vulnerability of patients with acute leukemia, in whom chemotherapy-related neurotoxicity, recurrent severe thrombocytopenia, systemic inflammation, and heightened metabolic demands may collectively amplify cerebral injury (15–17). Prior evidence indicates that even in the absence of macroscopic hemorrhage, these patients exhibit measurable declines in processing speed and executive function during intensive treatment (17). When an ICH occurs on this compromised substrate, cognitive impairment may become both more frequent and more consequential. Second, our study formally tested cognition as a mediator, moving beyond simple descriptions of co-occurring deficits. Although earlier studies have reported independent associations of neurological disability and cognitive scores with oncologic outcomes, none have partitioned the direct and cognitively mediated pathways (17, 18). This gap has left an incomplete understanding of why neurological disability disrupts oncologic care.

Our demonstration of a substantial indirect effect raises the question of how neurological deficits translate into cognitive decline and why cognitive decline, in turn, leads to treatment discontinuation. Both links are biologically and behaviorally plausible. The neurological deficits captured by the mRS at discharge predominantly reflect focal sensorimotor injury from the hemorrhage (19, 20); however, the same subcortical and periventricular brain regions that cause motor disability often intersect with white matter tracts subserving attention, memory, and executive control (21). Consequently, more severe ICH is expected to produce both higher mRS scores and, through diaschisis and secondary neurodegeneration, progressive cognitive decline (22, 23), a trajectory supported by longitudinal neuroimaging studies showing accelerated cortical atrophy and leukoaraiosis progression following deep ICH (23–25). The six-month cognitive assessment window we employed captures this evolving cognitive injury at a clinically relevant juncture when decisions regarding consolidation chemotherapy and transplantation candidacy are being finalized. This neuroanatomical and temporal convergence implies that the mRS, though a gross measure of disability, indirectly indexes a broader burden of brain injury that manifests behaviorally as cognitive decline. This decline, in turn, compromises the very abilities needed to sustain intensive treatment. Cognitive impairment can disrupt hematologic treatment through at least three interconnected routes: (i) executive dysfunction reduces a patient's capacity to adhere to complex outpatient regimens, attend frequent appointments, and recognize early signs of complications; (ii) impairments in memory and processing speed undermine informed consent and shared decision-making, especially when treatment options carry significant risk; and (iii) clinicians' subjective perception of cognitive frailty, even independent of objective scores, may bias them toward less intensive regimens—a phenomenon well documented in geriatric oncology but newly relevant in this context.

Our findings must also be weighed against existing knowledge on determinants of treatment discontinuation in acute leukemia. Refractory or progressive disease, severe infections, and poor overall performance status are recognized contributors to early treatment termination in this population (4); however, these factors were systematically excluded or adjusted for in our design, as we removed patients whose termination was solely due to disease progression and controlled for hematoma volume and leukemia type. The substantial total effect of mRS on discontinuation (HR 3.42) corroborates and quantifies what has been clinically suspected: that neurological morbidity independently drives early cessation of curative therapy. What our mediation analysis adds is the insight that a sizeable component of this risk is not directly attributable to irreversible focal motor or sensory deficits (such as hemiplegia), but rather to the cognitive consequence that may, at least theoretically, be amenable to intervention. This distinction is clinically meaningful because it reframes cognitive impairment from a passive comorbidity to an active mediator of oncologic failure. It should be noted, however, that the natural direct effect encompasses all pathways not operating through the measured cognitive score, including unmeasured behavioral, psychological, and social mechanisms (26). Some of these, such as post-ICH depression or loss of caregiver support (26, 27), may themselves be modifiable, and their contribution to the direct pathway warrants investigation in future studies.

Several methodological strengths support the validity of our findings. First, we applied causal mediation analysis for time-to-event data with robust bootstrapping and E-value quantification, an advance over conventional approaches that ignore the mediator or fail to establish temporal ordering. Second, restricting the cohort to patients alive and on treatment at six months enforced the exposure–mediator–outcome sequence required for valid causal inference. Third, sensitivity analyses confirmed that results were consistent across alternative modeling choices and unlikely to be fully explained by plausible unmeasured confounding. Finally, treatment discontinuation reasons were independently adjudicated by hematologists blinded to cognitive status, minimizing ascertainment bias.

Nonetheless, several limitations must be acknowledged. First, conditioning on 6-month survival and continued treatment restricts the cohort to a ‘principal stratum' of more resilient patients. Because neurological deficit severity itself influences early mortality and early treatment cessation, this conditioning induces selection (collider) bias: patients with severe deficits who nonetheless survived and remained on treatment for six months may differ systematically from those who died or discontinued earlier. The estimated effects are therefore conditional on the survivor-and-adherent stratum and cannot be generalized to the entire population of patients with acute leukemia and ICH. Second, the retrospective design precludes exclusion of residual confounding. Several prognostically relevant variables were not reliably captured in the medical record, including depression and other post-ICH emotional outcomes, functional dependence beyond that captured by the mRS, socioeconomic status and financial barriers to care, caregiver availability and support, frailty, formal performance status (ECOG/KPS), and the intensity of post-ICH hematologic treatment. Each of these could plausibly influence both cognition and treatment discontinuation, and although the E-value (2.66) provides partial reassurance against unmeasured mediator-outcome confounding, it cannot address all of these pathways simultaneously. Third, the sample of 102 patients, with only 45 discontinuation events, is small for a mediation analysis involving multiple covariates, bootstrap estimation, and E-value calculation. This limits statistical power for subgroup and interaction analyses and raises the possibility of unstable estimates and overfitting; the wide confidence interval for the proportion mediated (15.4%–68.4%) reflects this imprecision, and the point estimates should therefore be interpreted with appropriate caution. Fourth, our estimates may be biased by violation of assumption A4 (no exposure-induced mediator–outcome confounding). Post-discharge depression, caregiver loss, or intercurrent illness-potentially triggered by the neurological deficit-could simultaneously worsen cognition and treatment persistence, yet remained unmeasured; this is arguably the most consequential threat to causal interpretation, because preserving cognition would not necessarily prevent discontinuation if the same post-exposure factors drive it. Unlike the E-value, which addresses pre-exposure confounding, this post-exposure pathway is inherently untestable in our data. Fifth, a potential circularity exists between the mediator (MoCA-defined cognitive impairment) and the outcome definition, insofar as “cognitive inability to comply” was one adjudicated reason for discontinuation; although the cognitive score and the adjudicated reason derive from different information sources, this overlap warrants caution and motivated a corresponding sensitivity analysis (Section 3.4). Sixth, as a single-center retrospective study from one institution in China, external validity is limited; practice patterns, treatment intensity, and cognitive screening thresholds may differ across centers and health systems, and our findings require replication in multicenter cohorts. Seventh, the MoCA provides only a global cognitive measure; domain-specific neuropsychological testing is needed to identify which cognitive functions drive the mediation. Eighth, treatment discontinuation decisions, though independently adjudicated, may reflect incompletely documented patient and physician preferences. Finally, systematic data on cognitive rehabilitation were unavailable.

Despite these limitations, our findings have direct clinical and research implications. Cognitive impairment mediated 42.1% of the excess treatment discontinuation risk, suggesting that preserving cognitive function could substantially improve hematologic outcomes. First, routine cognitive screening may warrant integration into post-ICH care for leukemia patients. Second, our findings support the rationale for randomized trials testing whether early cognitive rehabilitation, initiated before the six-month treatment decision point, could reduce discontinuation rates. Cognitive interventions have shown modest efficacy in neuro-oncologic and traumatic brain injury populations, yet remain unstudied in acute leukemia. Third, hematologists and neurologists should jointly develop shared decision-making protocols that account for cognitive limitations, involving caregivers and neuropsychologists when critical treatment choices arise.

## 5 Conclusions

In this retrospective cohort of acute leukemia survivors with ICH, cognitive impairment at six months was estimated to mediate 42.1% of the total effect of neurological deficit severity on hematologic treatment discontinuation. This indirect pathway was consistent across multiple sensitivity analyses. Our findings suggest that post-hemorrhagic cognitive dysfunction may be not merely a concomitant morbidity but a potentially modifiable contributor through which structural brain injury interrupts curative oncologic therapy. Cognitive screening and early rehabilitation may therefore warrant integration into post-ICH care in this population. Whether cognitive interventions can truly modify this pathway remains to be prospectively tested.

## Data Availability

The raw data supporting the conclusions of this article will be made available by the authors, without undue reservation.

## References

[1] Hu J Lai W. Exploration of neuroprotective strategies in patients with acute promyelocytic leukemia complicated by cerebral hemorrhage. Front Med (Lausanne). (2025) 12:1597070. 10.3389/fmed.2025.1597070 40823554 PMC12350354

[2] Gi T Kai K Shide K Nakamura E Oguri N Aman M. Cathepsin G is associated with cerebral vascular injury in myeloid leukemia: a pathologic insight into intracranial hemorrhage. Res Pract Thromb Haemost. (2026) 10(3):103433. 10.1016/j.rpth.2026.103433 42023399 PMC13098595

[3] Shi L Yin P Chen C Fan Q Sun C Wang D. Machine learning-based model for predicting outcomes in cerebral hemorrhage patients with leukemia. Eur J Radiol. (2024) 177:111543. 10.1016/j.ejrad.2024.111543 38905800

[4] Jabbour E Short NJ Senapati J Jain N Huang X Daver N. Mini-hyper-CVD plus inotuzumab ozogamicin, with or without blinatumomab, in the subgroup of older patients with newly diagnosed Philadelphia chromosome-negative B-cell acute lymphocytic leukaemia: long-term results of an open-label phase 2 trial. Lancet Haematol. (2023) 10(6):e433–44. 10.1016/S2352-3026(23)00073-X 37187201 PMC11840755

[5] Rahiman EA Rajendran A Sankhyan N Singh P Muralidharan J Bansal D. Acute neurological complications during acute lymphoblastic leukemia therapy: a single-center experience over 10 years. Indian J Cancer. (2021) 58(4):545–52. 10.4103/ijc.IJC_422_19 34380827

[6] Rost NS Brodtmann A Pase MP Van Veluw SJ Biffi A Duering M. Post-stroke cognitive impairment and dementia. Circ Res. (2022) 130(8):1252–71. 10.1161/CIRCRESAHA.122.319951 35420911

[7] Kazim SF Ogulnick JV Robinson MB Eliyas JK Spangler BQ Hough TJ. Cognitive impairment after intracerebral hemorrhage: a systematic review and meta-analysis. World Neurosurg. (2021) 148:141–62. 10.1016/j.wneu.2021.01.026 33482414

[8] Shi X Bai H Wang J Wang J Huang L He M. Behavioral assessment of sensory, motor, emotion, and cognition in rodent models of intracerebral hemorrhage. Front Neurol. (2021) 12:667511. 10.3389/fneur.2021.667511 34220676 PMC8248664

[9] Li ZQ Bu XQ Cheng J Deng L Lv XN Wang ZJ. Impact of early cognitive impairment on outcome trajectory in patients with intracerebral hemorrhage. Ann Clin Transl Neurol. (2024) 11(2):368–76. 10.1002/acn3.51957 38009388 PMC10863917

[10] Simeoni M Mulholland MM Workeneh BT Capasso A Hafez G Liabeuf S. Chemotherapy-related cognitive impairment and kidney dysfunction. Nephrol Dial Transplant. (2025) 40(Supplement_2):ii54–63. 10.1093/ndt/gfae249 40080088 PMC11997771

[11] Kuil LE Nicola C Dietrich J Castel H. Neurotoxicity of chemotherapy and brain-radiotherapy in cancer: overlapping mechanisms underlying cognitive impairment. J Clin Exp Neuropsychol. (2025) 47(8):806–46. 10.1080/13803395.2025.2539199 40784023

[12] Zhang JY Li Y Ma YS Sun XJ Liu YZ Yin YK. Clinical characteristics and prognostic factors in intracranial hemorrhage patients with hematological diseases. Ann Hematol. (2022) 101(12):2617–25. 10.1007/s00277-022-04982-w 36178488 PMC9646537

[13] Valeri L Vanderweele TJ. Mediation analysis allowing for exposure-mediator interactions and causal interpretation: theoretical assumptions and implementation with SAS and SPSS macros. Psychol Methods. (2013) 18(2):137–50. 10.1037/a0031034 23379553 PMC3659198

[14] Vanderweele TJ Vansteelandt S. Odds ratios for mediation analysis for a dichotomous outcome. Am J Epidemiol. (2010) 172(12):1339–48. 10.1093/aje/kwq332 21036955 PMC2998205

[15] Gandy K Scoggins MA Jacola LM Litten M Reddick WE Krull KR. Structural and functional brain imaging in long-term survivors of childhood acute lymphoblastic leukemia treated with chemotherapy: a systematic review. JNCI Cancer Spectr. (2021) 5(5):pkab069. 10.1093/jncics/pkab069 34514328 PMC8421809

[16] Cheung YT To KK Hua R Lee CP Chan AS Li CK. Association of markers of inflammation on attention and neurobehavioral outcomes in survivors of childhood acute lymphoblastic leukemia. Front Oncol. (2023) 13:1117096. 10.3389/fonc.2023.1117096 37416531 PMC10320851

[17] Chan Y-N Betancur S Conklin JL Hirschey R Piepmeier A Foster M. Cognitive function in adults with acute myeloid leukemia treated with chemotherapy: a systematic review. Cancer Nurs. (2024) 47(2):121–31. 10.1097/NCC.0000000000001164 36066343 PMC10232672

[18] Cheung YT Sabin ND Reddick WE Bhojwani D Liu W Brinkman TM. Leukoencephalopathy and long-term neurobehavioural, neurocognitive, and brain imaging outcomes in survivors of childhood acute lymphoblastic leukaemia treated with chemotherapy: a longitudinal analysis. Lancet Haematol. (2016) 3(10):e456–66. 10.1016/S2352-3026(16)30110-7 27658980 PMC5417340

[19] Li N Guo J Kang K Zhang J Zhang Z Liu L. Cytotoxic edema and adverse clinical outcomes in patients with intracerebral hemorrhage. Neurocrit Care. (2023) 38(2):414–21. 10.1007/s12028-022-01603-2 36180765 PMC10090026

[20] Chen J Sheng D Liu X Ye X Deng X Sheng Y. Chemokines-like factor 1 as a serological biomarker in relation to disease severity and 90-day prognosis after spontaneous intracerebral hemorrhage: a prospective cohort study. Neurosurg Rev. (2025) 48(1):430. 10.1007/s10143-025-03602-1 40394360

[21] Șalari G Slevin M. mCRP-Associated vascular pathophysiology in progression and outcome of intracerebral hemorrhage. Int J Mol Sci. (2025) 26(13):6195. 10.3390/ijms26136195 PMC12249605 40649973

[22] Egorova-Brumley N Dhollander T Khan W Khlif MS Ebaid D Brodtmann A. Changes in white matter microstructure over 3 years in people with and without stroke. Neurology. (2023) 100(16):e1664–72. 10.1212/WNL.0000000000207065 36792378 PMC10115498

[23] Kuohn LR Witsch J Steiner T Sheth KN Kamel H Navi BB. Early deterioration, hematoma expansion, and outcomes in deep versus lobar intracerebral hemorrhage: the FAST trial. Stroke. (2022) 53(8):2441–8. 10.1161/STROKEAHA.121.037974 35360929

[24] Morotti A Li Q Nawabi J Mazzacane F Schlunk F Shoamanesh A. Volume tolerance and prognostic impact of hematoma expansion in deep and lobar intracerebral hemorrhage. Stroke. (2025) 56(5):1224–31. 10.1161/STROKEAHA.124.049008 40109238 PMC12037302

[25] Hillal A Apostolaki-Hansson T Ramgren B Norrving B Hansen B Wassélius J. Imaging and clinical prognostic features in lobar versus deep intracerebral hemorrhage in an unselected Swedish population. Eur J Neurol. (2025) 32(8):e70318. 10.1111/ene.70318 40760859 PMC12322319

[26] Ozkan H Ambler G Banerjee G Mitchell JJ Barbato C Browning S. Prevalence, predictors, and patterns of patient reported non-motor outcomes six months after stroke: a prospective cohort study. Lancet Reg Health Eur. (2024) 47:101080. 10.1016/j.lanepe.2024.101080 39498117 PMC11532962

[27] Lester EG Fishbein NS Higgins O Rosand J Vranceanu AM. Understanding the interplay between lifestyle factors and emotional distress for hemorrhagic stroke survivors and their informal caregivers: protocol for a mixed methods dyadic natural history study. PLoS One. (2022) 17(1):e0261635. 10.1371/journal.pone.0261635 35061739 PMC8782334

