# Supplementary material for: Cognitive impairment as a mediator of treatment discontinuation after intracerebral hemorrhage in acute leukemia
Source: Front Med (Lausanne). 2026 Sep 7;13:1928027. doi: 10.3389/fmed.2026.1928027 (PMC13590375; doi:10.3389/fmed.2026.1928027)
Supplement: Supplementary file 1 [file Table1.docx]

Table S1. Parameter Estimates from the Mediator (Linear Regression) and Outcome (Cox Proportional Hazards) Models Used in the Primary Causal Mediation Analysis.

Panel A. Mediator Model – Linear Regression Predicting MoCA Total Score at 6 Months (T1)

| Predictor | β (SE) | 95% CI | P value |
| --- | --- | --- | --- |
| Intercept | 28.42 (2.10) | 24.30, 32.54 | <0.001 |
| Neurological deficit: mRS 3–5 (vs. 0–2) | −5.86 (0.81) | −7.45, −4.27 | <0.001 |
| Age (per 1-year) | −0.08 (0.03) | −0.14, −0.02 | 0.008 |
| Education (per 1-year) | 0.41 (0.12) | 0.17, 0.65 | 0.001 |
| Hematoma volume (per 1 mL) | −0.04 (0.02) | −0.08, −0.001 | 0.045 |
| Leukemia type: ALL (vs. AML) | −0.72 (0.95) | −2.58, 1.14 | 0.45 |
| Treatment phase at ICH |  |  |  |
| Consolidation (vs. Induction) | 0.33 (0.92) | −1.48, 2.14 | 0.72 |
| Maintenance (vs. Induction) | −1.15 (1.42) | −3.94, 1.64 | 0.42 |

*Adjusted R² = 0.52.*

Note: The coefficient for mRS 3–5 represents the adjusted mean difference in MoCA score between the moderate-to-severe and mild deficit groups (−5.86), consistent with the unadjusted difference of −5.9 reported in Table 2.

Panel B. Outcome Model – Cox Proportional Hazards for Time to Hematologic Treatment Discontinuation (T2)

| Predictor | β (log-HR) | HR | 95% CI | P-value |
| --- | --- | --- | --- | --- |
| Neurological deficit: mRS 3–5 (vs. 0–2) | 0.732 | 2.08 | 1.13 – 3.84 | 0.014 |
| MoCA total score (per 1-point increase) | −0.094 | 0.91 | 0.87 – 0.96 | < 0.001 |
| Age (per 1-year) | 0.020 | 1.02 | 0.99 – 1.04 | 0.16 |
| Education (per 1-year) | −0.062 | 0.94 | 0.88 – 1.01 | 0.08 |
| Hematoma volume (per 1 mL) | 0.010 | 1.01 | 0.99 – 1.02 | 0.34 |
| Leukemia type: ALL (vs. AML) | 0.285 | 1.33 | 0.67 – 2.65 | 0.41 |
| Treatment phase at ICH |  |  |  |  |
| Consolidation (vs. Induction) | −0.198 | 0.82 | 0.40 – 1.66 | 0.58 |
| Maintenance (vs. Induction) | 0.140 | 1.15 | 0.43 – 3.08 | 0.78 |

Likelihood ratio test: χ² = 52.3, df = 8, P < 0.001.

Note: The HR for mRS 3–5 (2.08) corresponds to the natural direct effect reported in Table 3. The proportional hazards assumption was met for all variables (P > 0.10 for Schoenfeld residual tests).
